# AD|ARC (Administrative Data Agri-Research Collection): Linking Individual, Household and Farm Business Data for Agricultural Research.

**DOI:** 10.23889/ijpds.v7i3.1890

**Published:** 2022-08-25

**Authors:** Paul Caskie, Sian Morrison-Rees

**Affiliations:** 1 Agri-Food and Bioscience Institute (AFBI) Northern Ireland; 2 Pediatric Department, Hospital Lillebaelt Kolding

## Objectives

To create an anonymised data resource of farm households in the UK to generate evidence to support policy development, implementation and evaluation; improve understanding of farm family socio-economic characteristics; and assist all stakeholders interested in understanding about the health and well-being, resilience and prosperity and spatial properties of farming communities.

## Approach

The AD|ARC resource is being created in each UK country. The core dataset comprises of linking together existing agricultural datasets (farm business activity data, Rural Payments data) the Inter Departmental Business Register and the Population Census. This hub dataset is linked to the health and education data available in each country. The research is being conducted in a separate but coordinated way across the UK, harmonising the data to allow aggregation to a UK level where possible.

Our research objectives are in four broad categories of Socio-Demographics, Health and Well-being, Prosperity and Resilience and Environment and Place.

## Results

The presentation will report on initial findings from the socio-demographic research theme where researchers have investigated the composition and characteristics of farming households both in terms of the farm business (e.g. farm type, turnover, active farm diversification) and individual characteristics (e.g. age, educational attainment, off-farm income streams). From this analysis it will be possible to determine the degree of homogeneity that exists within the farming population for key indicators, the degree of homogeneity that exists amongst the broader group of farm households members for key indicators, and the extent to which farmers and farm household members conform to the socio-economic characteristics of the non-farming rural population. Results will be assessed for policy relevance and areas of ongoing investigation will be highlighted.

## Conclusion and Relevance

Agriculture is currently facing a range of challenges with little known about the impact on farmers and farming households. AD|ARC introduces a new, powerful and versatile resource that will inform debate and policy decision-makers, potentially leading to better outcomes on a range of issues relevant to farmers and farming communities.

